# Boosting chemokine receptor recycling: an elixir of life for chronic lymphocytic leukemia

**DOI:** 10.18632/oncotarget.26099

**Published:** 2018-09-11

**Authors:** Laura Patrussi, Cosima T. Baldari

**Affiliations:** Laura Patrussi: Department of Life Sciences, University of Siena, Siena, Italy

**Keywords:** recycling, chronic lymphocytic leukemia, chemokine receptor, p66Shc, calcineurin

The stromal microenvironment of lymphoid organs is emerging as a key player in regulating both survival and chemoresistance of chronic lymphocytic leukemia (CLL) cells [1]. Abnormally elevated surface levels of the homing receptors CCR7 and CXCR4 [2], and defective expression of the egress receptor S1PR1 [3], alter the balance between entry to and exit from lymphoid organs, prolonging leukemic cell residency in this pro-survival niche.

In addition to transcriptional modulation, the surface amount of chemokine receptors is regulated by endosome recycling, which allows for rapid re-use of engaged receptors without need of new synthesis. Enhanced transcription and potentiated recycling cooperate to alter the chemokine receptor landscape in CLL cells, eventually impacting on leukemic cell residency in the protective lymphoid niche. Interestingly, while CCR7 exploits both transcription and recycling to upregulate its surface levels in CLL cells [2, 3], CXCR4 completely relies on enhanced recycling [2]. This finding underlies the importance of this mechanism and the serious effects of its imbalance in pathological contexts related to altered cell migration.

We have reported that a defect in the expression of the pro-apoptotic adaptor p66Shc in CLL cells [4] contributes to CLL pathogenesis by enhancing CCR7 transcription while concomitantly decreasing S1PR1 expression [2], thereby promoting leukemic cell accumulation in the stromal environment. In our recent report [5], based on the ability of p66Shc to negatively modulate chemokine receptor signaling [6], we asked whether the p66Shc defect is also involved in enhanced recycling of CXCR4 and CCR7 in CLL cells. Consistent with this hypothesis, results obtained using the CLL-derived cell line MEC which does not express p66Shc [3] transfected with a p66Shc-encoding vector, or B cells isolated from p66Shc^-/-^ and wild-type mice, demonstrated a negative impact of p66Shc on CXCR4 and CCR7 recycling. Additionally, the abnormalities in CXCR4 and CCR7 recycling in CLL cells could be rescued by forced p66Shc expression. Hence the p66Shc defect of CLL cells translates into their enhanced ability to recycle these receptors.

**Figure 1:**
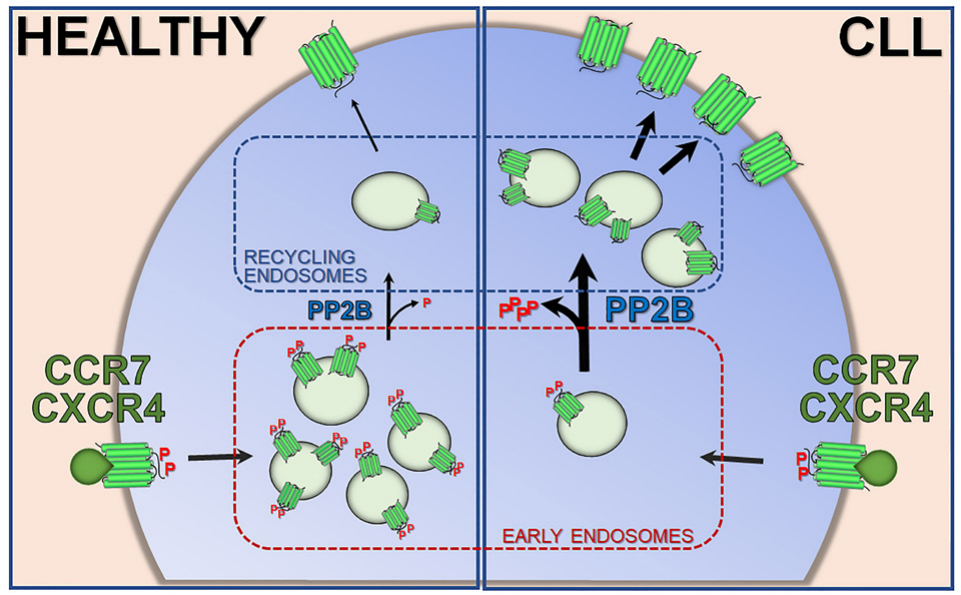
Enhanced CXCR4 and CCR7 recycling in CLL B cells Schematic representation of enhanced recycling of CXCR4 and CCR7 homing receptors in normal B cells (HEALTHY) and in leukemic cells from CLL patients (CLL). EE: early endosomes; RE: recycling endosomes; P: serine/threonine phosphorylation.

To dissect the molecular circuitry that connects p66Shc to chemokine receptor recycling we tracked CXCR4 and CCR7 undergoing recycling in leukemic B cells. We found that both become phosphorylated, recruit β-arrestin and become internalized in Rab5^+^ early endosomes, a sorting compartment wherefrom internalized receptors are directed either to recycling endosomes to return to the plasma membrane or to late endosomes for their subsequent lysosome-mediated degradation. Interestingly, we observed a change in the intracellular distribution of CXCR4 and CCR7 in CLL cells, with a preferential accumulation in Rab11^+^ recycling endosomes. This was paralleled by an expansion of the surface pool of either receptor at the expense of the intracellular pool. Interestingly, forced p66Shc expression in these cells reverted these abnormalities, indicating that p66Shc affects the subcellular distribution of CXCR4 and CCR7 by slowing down their transit to the endosomal recycling compartment, ultimately restraining the surface pool of these receptors (Figure 1).

Having verified that that receptor internalization was not affected by p66Shc, we focused on the process of receptor dephosphorylation, that allows for β-arrestin release and receptor access to the recycling route. Immunoprecipitation experiments and flow cytometric analyses of the phosphorylated receptors in the MEC transfectants as well as in CLL cells showed that both receptors were readily dephosphorylated in cells lacking p66Shc, consistent with their rapid re-exposure at the plasma membrane, while their phosphorylation was significantly delayed in p66Shc-overexpressing cells. Using pharmacological inhibitors and siRNA-mediated knock-down were able to identify PP2B, broadly known as Calcineurin, as the phosphatase responsible for dephosphorylating CXCR4 and CCR7. The activity of PP2B crucially relies on [Ca^2+^]_i_. The ability of p66Shc to attenuate [Ca^2+^]_i_ mobilization in response to CXCR4 and CCR7 engagement provides mechanistic insights into the inverse correlation between p66Shc expression and PP2B activity as well as receptor recycling.

We were intrigued by the fact that the Btk inhibitor ibrutinib, used for the treatment of CLL, potently mobilizes leukemic cells from lymph nodes [7]. Our previous results provided evidence that ibrutinib promotes leukemic cell exit from the stromal niche by normalizing the balance between CCR7 and S1PR1 [2]. We now add a tile to the puzzle by demonstrating that ibrutinib operates by enhancing p66Shc expression in CLL cells, which importantly translates into the intracellular accumulation of CXCR4/CCR7 as a consequence of their inability to transit along the recycling route. Reconstitution of p66Shc expression in CLL B cells appears therefore of interest for the treatment of this as yet incurable disease. Although ibrutinib is already used for CLL treatment, its specific molecular target is Btk, confining the p66Shc-elevating ability to a side-effect. The development of drugs able to specifically enhance p66Shc expression, e.g. by activating the p66Shc transcription factor STAT4 [8], might be a potential new frontier for the treatment of CLL.

To our knowledge our report is the only one to date to have directly addressed the possibility that alterations in the vesicular traffic pathway responsible for chemokine receptor recycling might underlie the abnormalities in surface expression of CXCR4 and CCR7 and the resulting enhancement in leukemic cell homing to the pro-survival stromal niche [5]. Dissecting this pathway may unveil other molecular players that are modulated in CLL and that could be amenable to pharmacological manipulation for new personalized therapies.
